# Thoracic motion‐compensated cone‐beam computed tomography in under 20 seconds on a fast‐rotating linac: A simulation study

**DOI:** 10.1002/acm2.13909

**Published:** 2023-01-21

**Authors:** Samuel J. Blake, Owen Dillon, Hilary L. Byrne, Ricky T. O'Brien

**Affiliations:** ^1^ ACRF Image X Institute, Sydney School of Health Sciences, Faculty of Medicine and Health The University of Sydney Sydney New South Wales Australia; ^2^ Medical Radiations, School of Health and Biomedical Sciences RMIT University Bundoora Victoria Australia

**Keywords:** computer simulation, cone‐beam computed tomography, image reconstruction, organ motion, radiotherapy

## Abstract

**Background:**

Rapid kV cone‐beam computed tomography (CBCT) scans are achievable in under 20 s on select linear accelerator systems to generate volumetric images in three dimensions (3D). Daily pre‐treatment four‐dimensional CBCT (4DCBCT) is recommended in image‐guided lung radiotherapy to mitigate the detrimental effects of respiratory motion on treatment quality.

**Purpose:**

To demonstrate the potential for thoracic 4DCBCT reconstruction using projection data that was simulated using a clinical rapid 3DCBCT acquisition protocol.

**Methods:**

We simulated conventional (1320 projections over 4 min) and rapid (491 projections over 16.6 s) CBCT acquisitions using 4D computed tomography (CT) volumes of 14 lung cancer patients. Conventional acquisition data were reconstructed using the 4D Feldkamp‐Davis‐Kress (FDK) algorithm. Rapid acquisition data were reconstructed using 3DFDK, 4DFDK, and Motion‐Compensated FDK (MCFDK). Image quality was evaluated using Contrast‐to‐Noise Ratio (CNR), Tissue Interface Width (TIW), Root‐Mean‐Square Error (RMSE), and Structural SIMilarity (SSIM).

**Results:**

The conventional acquisition 4DFDK reconstructions had median phase averaged CNR, TIW, RMSE, and SSIM of 2.96, 8.02 mm, 83.5, and 0.54, respectively. The rapid acquisition 3DFDK reconstructions had median CNR, TIW, RMSE, and SSIM of 2.99, 13.6 mm, 112, and 0.44 respectively. The rapid acquisition MCFDK reconstructions had median phase averaged CNR, TIW, RMSE, and SSIM of 2.98, 10.2 mm, 103, and 0.46, respectively. Rapid acquisition 4DFDK reconstruction quality was insufficient for any practical use due to sparse angular projection sampling.

**Conclusions:**

Results suggest that 4D motion‐compensated reconstruction of rapid acquisition thoracic CBCT data are feasible with image quality approaching conventional acquisition CBCT data reconstructed using standard 4DFDK.

## 1 INTRODUCTION

Kilovoltage (kV) cone‐beam CT (CBCT) for pre‐treatment image guidance in radiotherapy is routine clinical practice in the treatment of several cancer sites. A recent international survey reported that kV imaging facilities were available on between 80% and 96% of conventional C‐arm medical linear accelerators (linacs) in most countries, with kV CBCT being the method most frequently used for image‐guided radiotherapy (IGRT).^1^ Furthermore, for several countries surveyed most radiotherapy centers used four‐dimensional (4D) CBCT for respiratory‐controlled treatment delivery.

Halcyon (Varian Medical Systems, Palo Alto, CA) is a compact ring‐mounted linac with integrated kV imaging system designed to streamline and improve access to IGRT technologies. Key features include gantry rotation up to four times faster than conventional linacs, an integrated kV x‐ray imaging system with increased field of view and frame rates up to 30 Hz, and fast kV CBCT acquisition with 11 pre‐set scanning protocols utilizing different numbers of projections and beam energies.^2^ A VersaHD linac (Elekta AB, Stockholm, Sweden) with kV CBCT imaging unit has also been modified under a research agreement to enable ultrafast gantry rotation up to 18 degrees per second, compared to the standard rotation speed of 3 degrees per second.^3^

Studies have reported on the characterization and implementation of Halcyon's kV x‐ray image guidance features.^2^, ^4^ Cai et al. characterized all kV CBCT protocols available on a preclinical Halcyon 2.0 system.^2^ Scans were completed in 17–42 s, compared to 60 s on a C‐arm linac. Peng et al. developed an automated process to compare the image quality of all kV CBCT protocols on the Halcyon and evaluated this process over a 10‐month period to assess image quality stability.^4^

Halcyon kV CBCT protocols are currently limited to 3D. However, 4DCBCT capabilities are desired for thoracic treatments to ensure that the target does not move outside the treatment volume. ESTRO‐ACROP consensus guidelines on stereotactic body radiotherapy for peripherally‐located early‐stage non‐small cell lung cancer (NSCLC) recommend daily pre‐treatment 4D volumetric image guidance.^5^ Respiratory‐correlated reconstruction^6^ and motion‐compensated reconstruction^7^, ^8^ enable phase‐resolved reconstruction of thoracic CBCT images by binning projections according to the respiratory phase during which they were acquired and reconstructing separate volumes for each phase.

The Halcyon Thorax Fast protocol acquires 491 projections in 16.6 s. While respiratory‐correlated reconstruction requires longer scan times (e.g. 4 min) for adequate image quality, motion‐compensated reconstruction improves image quality using 1‐min scan times.^7^, ^8^ Furthermore, motion compensation enables low dose (200‐projection) 4DCBCT images to be reconstructed with minimal impact relative to the quality of conventional (1320‐projection) scans.^9^, ^10^ We hypothesized that the data acquired for 3D reconstruction on rapid scanning systems like Halcyon could generate 4D motion‐compensated reconstructions of comparable quality. To demonstrate this, we simulated Halcyon‐style rapid CBCT scans based on the Thorax Fast protocol and reconstructed the projection data with and without motion compensation. Images were compared to reconstructions from simulated conventional CBCT acquisition data and ground‐truth real patient 4DCT images.

## 2 METHODS

**FIGURE 1:**
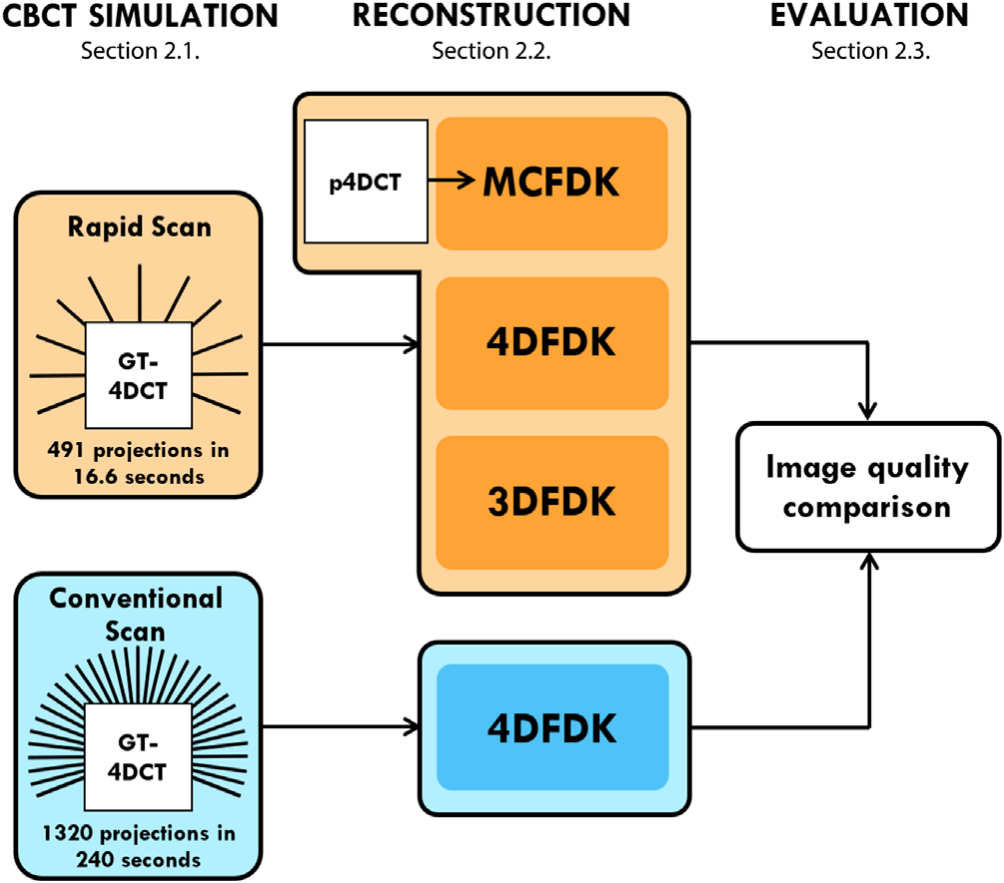
Study design schematic. The “ground‐truth” 4DCT (GT‐4DCT) volumes were used for simulating conventional and rapid CBCT acquisitions. Simulated projection data were reconstructed using three different algorithms: 3D and 4D Feldkamp‐Davis‐Kress (FDK) filtered backprojection and motion‐compensated FDK (MCFDK). The MCFDK algorithm used motion models derived from the “planning” 4DCT (p4DCT). Image quality metrics were computed for all reconstructions with the rapid acquisition reconstructions compared to the conventional acquisition 4DFDK reconstruction as baseline.

Figure 1 summarizes the study design. CBCT projection data were simulated using real patient “ground‐truth” 4DCT (GT‐4DCT) images according to conventional and rapid scan protocols (Section 2.1). Projection data were then reconstructed using three different algorithms (Section  2.2). Finally, rapid CBCT reconstructions were evaluated using several image quality metrics and compared to the conventional CBCT reconstruction as baseline (Section 2.3).

### 2.1 Cone‐beam CT simulations

The open‐source Reconstruction Tool Kit (RTK)^11^ was used to simulate conventional (1320 projections in 240 s) and rapid (491 projections in 16.6 s) 4DCBCT acquisitions over a 200‐degree arc by forward projecting through a 4DCT patient image. Patient data were obtained from The Cancer Imaging Archive^12^ 4D‐Lung dataset, which consists of 4DCT and 4DCBCT image data for 20 patients treated for locally advanced NSCLC.^13^, ^14^, ^15^, ^16^ Only 14 patients with repeat 4DCT scans were included in our study.

For each patient, a pair of 10‐phase 4DCT scans acquired on different days was used. The first was considered a “planning” 4DCT (p4DCT) that was used to derive an estimate of patient motion for select reconstruction algorithms (Section 2.B.2.). The second was a repeat scan considered as an up‐to‐date anatomical “ground‐truth” (GT‐4DCT) for CBCT simulation and image quality quantification.

The simulated CBCT scan geometry was full‐fan with a 1000 mm source‐to‐isocenter distance, 1536 mm source‐to‐detector distance and a 500 × 400 pixels detector with 1 mm^2^ pixels. Respiration was treated as regular with rates of 16.5 and 16.1 breaths per minute for the conventional and rapid scans, respectively, to ensure an integer number of projections allocated to each phase.

### 2.2 Image reconstruction algorithms

Simulated projection data were reconstructed using three different algorithms. All images were reconstructed with a 1×1×1 mm^3^ voxel size. Conventional acquisition data were reconstructed using the 4D Feldkamp‐Davis‐Kress (FDK) algorithm,^17^ the standard method for 4DCBCT reconstruction and the comparison baseline for this study. Rapid acquisition data were reconstructed using 3DFDK, 4DFDK, and motion‐compensated FDK (MCFDK). The same respiratory signal was used during CBCT simulation and for the 4DFDK and MCFDK reconstructions.

#### 2.2.1 3D and 4D FDK

Conventional and rapid acquisition data were reconstructed using the RTK implementation of the 4DFDK algorithm.^6^ Only the phase‐correlated projections were filtered and backprojected, reconstructing separate volumetric images for each respiratory phase. The rapid acquisition data were also reconstructed using the RTK implementation of the standard 3DFDK algorithm,^17^ which uses all projections to reconstruct a single volume.

#### 2.2.2 MCFDK

The rapid acquisition data were further reconstructed using the RTK implementation of the MCFDK algorithm.^7^ MCFDK uses all data in all respiratory phases by deforming the reconstruction of the projections from the phase they were acquired to a reference phase. By doing this, the projection under sampling problem that is inherent to 4DFDK reconstructions can be overcome.

More specifically, MCFDK differs from standard 4DFDK by using the p4DCT as an a priori model of the patient motion observed during CBCT acquisition. Nine of the p4DCT volumes are deformably registered to the tenth volume, considered as a reference volume and chosen to be peak inhale to maximize lung volume. This derives deformation vector fields (DVFs) to map each p4DCT volume to peak inhale. Once the CBCT is acquired, an initial rigid registration of a 3DFDK reconstruction to the prior 3DCT (average of the p4DCT volumes) is performed. This translation is applied to the p4DCT DVFs, which are then applied to the 4DFDK reconstructions of the CBCT, thus mapping each 4DFDK volume to peak inhale and enabling the warped volumes to be averaged to create a single reference MCFDK volume. Finally, the inverse DVFs were applied to the reference MCFDK volume to generate the remaining nine MCFDK volumes.

### 2.3 Evaluating reconstructed image quality

For each patient, all reconstructions described in Section 2.2 were evaluated in terms of contrast‐to‐noise ratio (CNR), tissue interface width (TIW), structural similarity index (SSIM) relative to the GT‐4DCT and root‐mean‐square error (RMSE) relative to the GT‐4DCT while considering the conventional acquisition 4DFDK reconstructions as baseline. Results for each reconstruction were compared to those of all other reconstructions using paired‐samples *t*‐tests to test for statistically significant differences. Metrics were quantified over subvolumes within the field of view to minimize the influence of truncation artefacts. Voxel values within these subvolumes were automatically affine windowed.^9^ One patient was excluded from the CNR and TIW analysis because the diaphragm was outside the field of view in most phases.

#### 2.3.1 Subvolume definition

**FIGURE 2:**
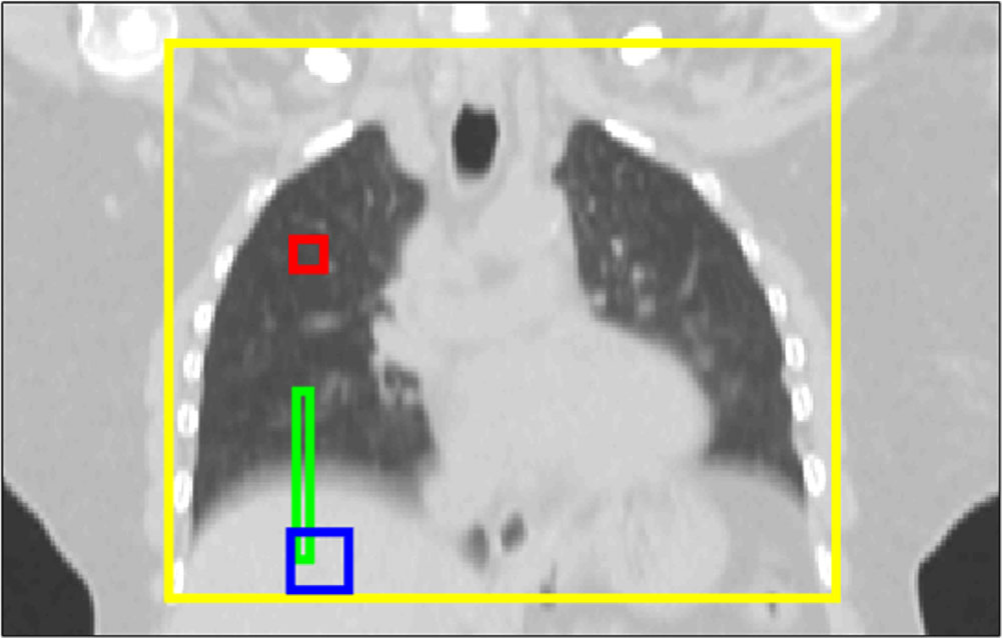
Coronal slice through a ground‐truth 3DCT for one patient with the subregions used to calculate CNR, TIW and SSIM highlighted in red/blue, green, and yellow, respectively.

Figure 2 shows a coronal slice through one patient's GT‐3DCT image (average of the GT‐4DCT) with the subvolumes highlighted to demonstrate how they were typically defined. All subvolumes were defined manually on each patient's GT‐3DCT image so that motion artefacts were visible during delineation. Histograms of pixel values within each subvolume were checked to ensure that anatomical deviations from the GT‐3DCT did not significantly impact the metrics being calculated. Subvolumes defined for calculating CNR were ensured to be approximately homogeneous while subvolumes defined for calculating TIW included the diaphragm and excluded substructures in the lung. Subvolumes for each patient were then copied onto all reconstructions and checked visually to ensure correct placement.

#### 2.3.2 Contrast‐to‐noise ratio (CNR)

The CNR measures differentiability of two tissues with similar contrast and was computed as

(1) $$\mathrm{CNR}=\frac{\left|\mu_{\mathrm{FG}}-\mu_{\mathrm{BG}}\right|}{\sigma }$$

where μ_FG_ and μ_BG_ are the mean voxel values from foreground (21 × 21 × 21 voxels) and background (11 × 11 × 11 voxels) subvolumes, respectively, and σ denotes standard deviation in the background subvolume. The foreground and background subvolumes (blue and red regions in Figure 2, respectively) were defined on the patient's right side in the liver and healthy upper lung, respectively.

#### 2.3.3 Tissue interface width (TIW)

The TIW is a measure of sharpness between the tissue and lung. We defined TIW as a more interpretable version of the tissue interface sharpness (TIS) metric^18^ where TIW represents the boundary width, with smaller values indicating sharper, more easily delineated images.

TIS was computed within a 5×5×l voxel subvolume defined at the superior border of the diaphragm, taking l=60 as the run length (green region in Figure 2). The 5×5=25 voxel runs in the superior‐inferior direction, each of length *l*, were extracted and normalized between 0 and 1. A sigmoid function was fit to each normalized voxel run, defining the TIS as the average sigmoid gradient. The TIW was then calculated as the physical distance across which the average sigmoid function assumes a value between 0.1 and 0.9, such that

(2) $$\mathrm{TIW}=\frac{2w\times \ln9}{\mathrm{TIS}}$$

where *w* is the reconstructed voxel length in mm.

#### 2.3.4 Root‐mean‐square error (RMSE) and structural similarity (SSIM)

The RMSE and SSIM measure similarity between two images with values closer to 0 and 1, respectively, indicating similar appearance or structure. Both were evaluated using the same subvolume (yellow region in Figure 2). The RMSE was computed as

(3) $$\mathrm{RMSE}=\frac{1}{\sqrt{n}}{\left|\left|x_{\mathrm{GT}}-x_{r}\right|\right|}_{2}$$

where *x*_GT_ and *x*_r_ denote the voxel value vectors within the GT‐4DCT and 4DCBCT reconstruction subvolumes, respectively, containing *n* voxels. SSIM was computed as

(4) $$\mathrm{SSIM}=\frac{\left(2\mu_{\mathrm{GT}}\mu_{r}+c_{1}\right)\left(2\sigma_{r,\mathrm{GT}}+c_{2}\right)}{\left(\mu_{\mathrm{GT}}^{2}+\mu_{r}^{2}+c_{1}\right)\left(\sigma_{\mathrm{GT}}^{2}+\sigma_{r}^{2}+c_{2}\right)}$$

where μ and σ^2^ denote voxel value means and variances, $c_{1}={\left(0.01L\right)}^{2}$ and $c_{2}={\left(0.03L\right)}^{2}$ where *L* is the dynamic range of the subvolumes. Subvolumes were defined to consistently fill as much of the thorax as possible within the field of view.

## 3 RESULTS

**FIGURE 3:**
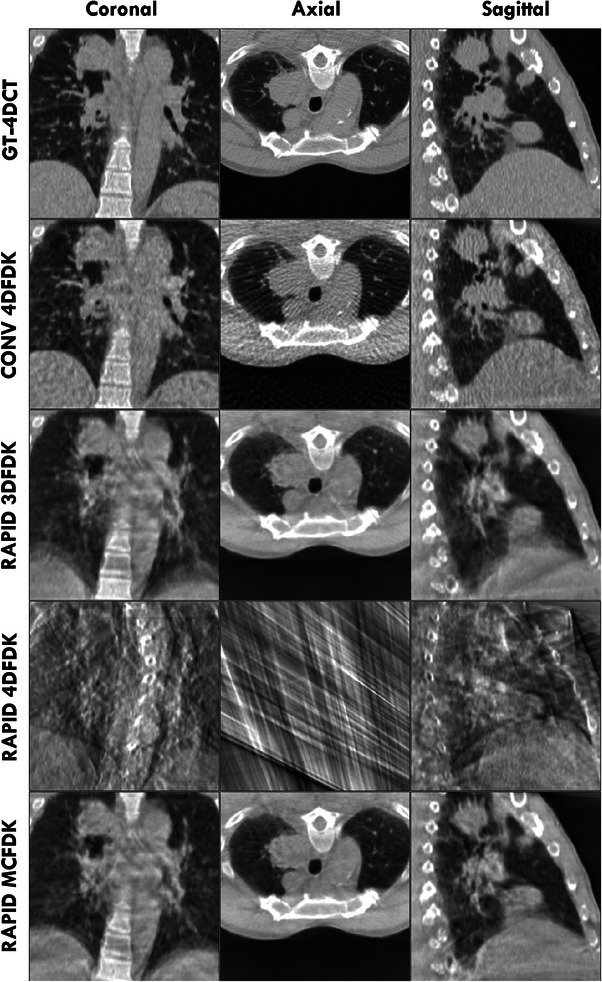
Coronal, axial and sagittal slice reconstructions through the tumor volume of the patient found to have the highest rapid acquisition MCFDK RMSE relative to the GT‐4DCT at peak exhale. The GT‐4DCT and each simulated scan acquisition/reconstruction method are shown. “CONV” indicates a conventional 1320 projection 4‐min acquisition while “RAPID” indicates a 491 projection 16.6‐s acquisition.

**FIGURE 4:**
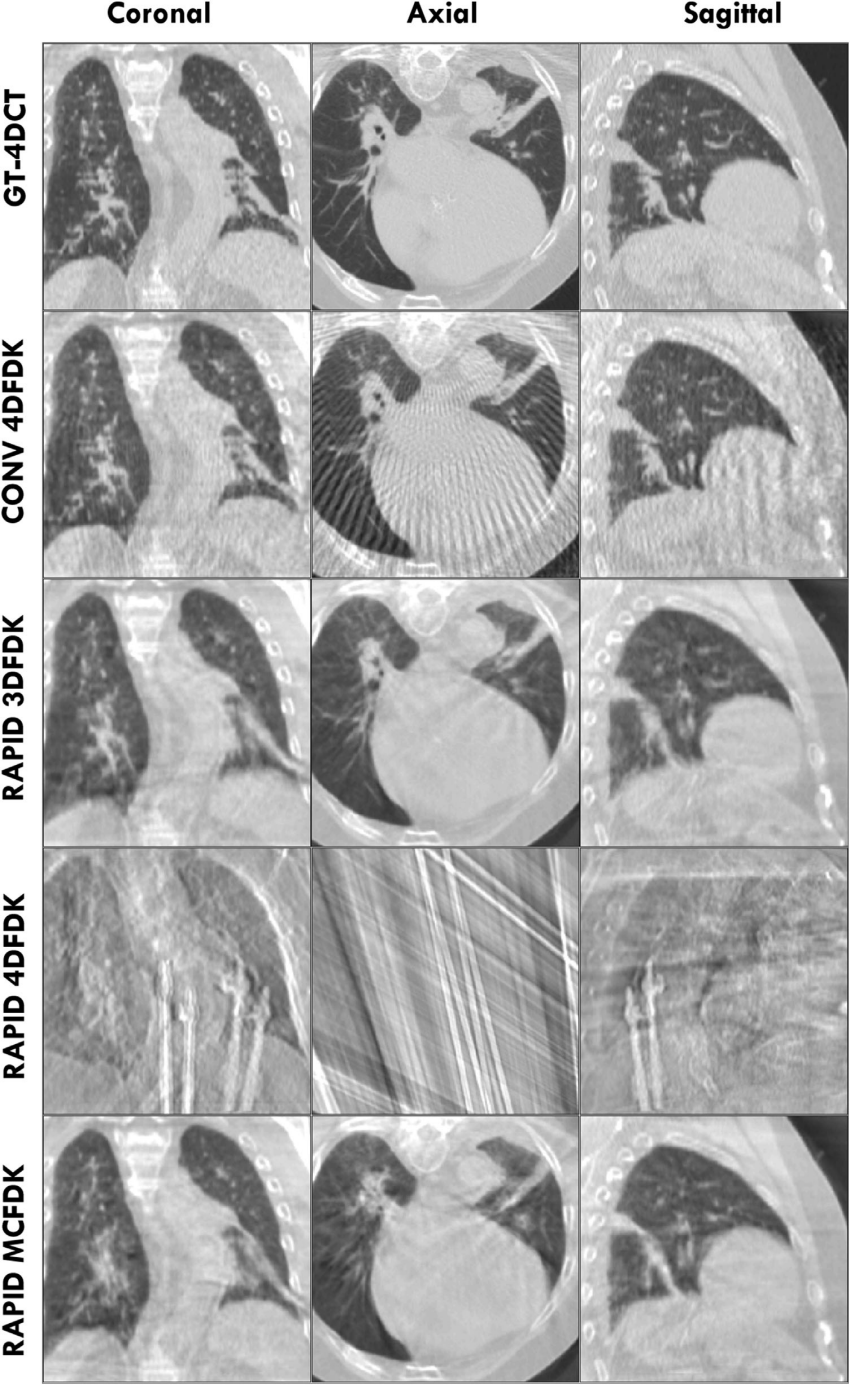
Coronal, axial and sagittal slice reconstructions through the tumor volume of the patient found to have the lowest rapid acquisition MCFDK RMSE relative to the GT‐4DCT at peak exhale. The GT‐4DCT and each simulated scan acquisition/reconstruction method are shown. “CONV” indicates a conventional 1320 projection 4‐min acquisition while “RAPID” indicates a 491 projection 16.6‐s acquisition.

Figures 3 and 4 show coronal, axial and sagittal slices through the GT‐4DCT, conventional acquisition 4DFDK, rapid acquisition 3DFDK, rapid acquisition 4DFDK and rapid acquisition MCFDK reconstructions for two sample patients at peak exhale. These patients had MCFDK reconstructions with the highest (Figure 3) and lowest (Figure 4) RMSE relative to the GT‐4DCT and are considered representative of the worst and best MCFDK reconstructions across the patient cohort, respectively.

Rapid acquisition 3DFDK reconstructions typically appeared smoother than conventional acquisition 4DFDK reconstructions. The streak artefacts present in the conventional 4DFDK reconstructions (clearest in the axial plane), resulting from the use of only the phase‐correlated projections to reconstruct each phase volume, were not visible in the rapid acquisition 3DFDK reconstructions. These artefacts were, however, dominant over the anatomical information in the 4DFDK reconstructions of the sparsely sampled rapid acquisition data. Consequently, rapid acquisition 4DFDK reconstruction quality was drastically inferior to that of the GT‐4DCT and all other CBCT reconstructions.

Rapid acquisition MCFDK reconstruction quality was generally comparable to that of the rapid acquisition 3DFDK and conventional acquisition 4DFDK reconstructions. The rapid acquisition MCFDK reconstructions and conventional acquisition 4DFDK reconstructions typically exhibited greater resolution and sharpness compared to the rapid acquisition 3DFDK reconstructions. The motion blur in the MCFDK reconstructions was generally more than that in the conventional acquisition 4DFDK reconstructions and less than that in the rapid acquisition 3DFDK reconstructions. Fine details, including cardiac substructures and vessels in the lungs, typically appeared sharpest in the conventional acquisition 4DFDK reconstructions.

**FIGURE 5:**
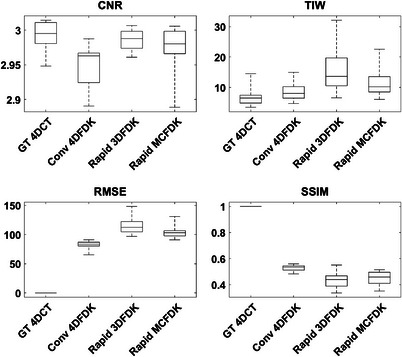
Boxplots of phase averaged contrast‐to‐noise ratio (CNR), tissue interface width (TIW), root‐mean‐square error (RMSE) and structural similarity index (SSIM) for the GT‐4DCT and each simulated scan acquisition and reconstruction method considered. Note that the rapid scan 4DFDK reconstruction data have been omitted as the image quality did not allow many of the metrics to be computed. For each box, the central line indicates the median value, the top and bottom edges of the box indicate the 75^th^ and 25^th^ percentiles, respectively and the whiskers extend to the maximum and minimum values. Image quality is generally better with increased CNR and SSIM, and decreased TIW and RMSE.

The quantitative image quality evaluation is summarized in Figure 5 as boxplots of the phase‐averaged CNR, TIW, RMSE, and SSIM for the GT‐4DCT images and for each simulated scan acquisition and reconstruction method considered. Note that results for the rapid acquisition 4DFDK reconstructions are not included in Figure 5 since reconstruction quality was insufficient to compute most metrics. Table 1 summarizes the *p‐*values calculated from paired‐samples *t*‐tests that were performed for each metric to test for statistically significant differences between pairs of reconstructions.

**TABLE 1:** *p*‐values from paired‐samples *t*‐tests comparing the differences in each image quality metric for the indicated pairs of reconstructions

	GT 4DCT—conv 4DFDK	GT 4DCT—rapid 3DFDK	GT 4DCT—rapid MCFDK	Conv 4DFDK—rapid 3DFDK	Conv 4DFDK—rapid MCFDK	Rapid 3DFDK—rapid MCFDK
CNR	**4.53 × 10^−7^**	0.062	**0.043**	**4.85 × 10^−5^**	**0.026**	0.116
RMSE	–	–	–	**1.04 × 10^−7^**	**8.74 × 10^−7^**	**3.40 × 10^−4^**
SSIM	–	–	–	**2.24 × 10^−5^**	**4.11 × 10^−5^**	0.157
TIW	0.002	**0.001**	0.009	**0.003**	0.055	**0.007**

Statistically significant differences were defined as those having p‐values less than 0.05 and are indicated using bold font.

Phase‐averaged CNR for all acquisition and reconstruction methods considered was generally within ∼3%. TIW, RMSE relative to GT and SSIM relative to GT were all typically worst for the rapid acquisition 3DFDK reconstructions. These metrics were generally comparable, or slightly worse, for the rapid acquisition MCFDK reconstructions when compared to the conventional acquisition 4DFDK reconstructions.

## 4 DISCUSSION

This study assessed the feasibility of motion compensated reconstruction of simulated rapid acquisition CBCT data and compared the quality of rapid acquisition reconstructions with and without motion compensation to conventional acquisition reconstructions.

We compared standard FDK and MCFDK reconstructions of rapid acquisition data because the former is standard‐of‐care for clinical 4DCBCT and the latter can generate images of comparable quality from 200 projections while maintaining clinically feasible computation times.^9^ Although MCFDK requires a prior image, typically the p4DCT, for motion compensation, these are readily available for most lung cancer patients treated with radiotherapy. The AAPM Task Group 324 respiratory motion management in radiation oncology survey found 4DCT as the most frequently used technique (93%) for simulation of thoracic cancer patients.^19^ A previous survey found in 2009 that 44% of centers had 4DCT available, with a growth rate of 6%–7% per year since 2003.^20^ Alternative reconstruction methods, including data driven approaches as in motion‐compensated McKinnon‐Bates (MKB)^9^, ^21^ and regularized iterative approaches as in ROOSTER,^22^ may be considered in future studies if improvements to computation time can be achieved.

The rapid acquisition 4DFDK reconstructions were excluded from quantitative analysis because of their drastically inferior quality. This was a consequence of performing standard 4DFDK reconstruction on the sparsely sampled rapid scan data where few respiratory cycles are captured throughout the scan duration. The normal breathing rate for a healthy adult is 16–25 breaths per minute (average 20),^23^ equivalent to ∼4–7 respiratory cycles per scan. While the impact of breathing rate on reconstruction quality was beyond the scope of this study, rates within this range will still result in sparsely sampled data from rapid acquisitions and thus are not expected to significantly affect 4DFDK reconstruction quality.

The CBCT reconstructions were compared quantitatively to the GT‐4DCT in terms of CNR, TIW, RMSE, and SSIM. All reconstructions had comparable CNR, however the rapid acquisition 3DFDK reconstructions generally performed worst in terms of TIW, RMSE relative to GT and SSIM relative to GT. This is consistent with the observation that the rapid acquisition 3DFDK reconstructions typically exhibited the most motion blur. The conventional acquisition 4DFDK reconstructions typically performed best in terms of TIW, RMSE relative to GT and SSIM relative to GT. Qualitatively this was most obvious when comparing the sharpness of substructures in the lungs and heart between the reconstruction methods. Resolving such fine detail is, however, typically not necessary for most common applications of CBCT in IGRT including patient setup verification.

The MCFDK reconstructions were quantitatively comparable though still worse than the conventional acquisition 4DFDK reconstructions. This reflects the fact that the MCFDK reconstructions used motion compensation derived from a prior p4DCT image, which will in general differ from motion contained within the GT‐4DCT used during simulation. When motion in the p4DCT differs from that in the GT‐4DCT, motion blur will persist in the MCFDK reconstructions. Given the short scan duration for rapid acquisitions, the impact of an inaccurate motion model was expected to be more detrimental to rapid acquisition reconstructions than conventional acquisition reconstructions. However, our observation that MCFDK reconstruction quality was generally comparable to that of conventional acquisition 4DFDK reconstructions is consistent with the findings of others.^7^, ^8^, ^10^ To assess whether the quality of a rapid acquisition MCFDK reconstruction is sufficient in practice, one could calculate the TIW across the diaphragm and accept the reconstruction if the TIW is sufficiently small. If the TIW is too large, a conventional acquisition CBCT could be acquired for improved quality. An alternative approach to motion compensation, motion‐compensated MKB,^9^ solves this problem by deriving motion estimates from data acquired on the day of treatment. Current implementations take ∼20 min to compute the MKB‐reconstruction and DVFs.^10^ This area needs further investigation to accelerate computation time so that motion‐compensated MKB may become clinically feasible.

The most computationally expensive part of MCFDK reconstruction was the DVF estimation from the p4DCT, taking approximately 2 h on our system. In practice this could be computed offline prior to 4DCBCT acquisition to not hinder clinical feasibility. The post‐4DCBCT acquisition steps include an initial rigid registration of the 3DFDK reconstruction to the prior 3DCT, application of this translation to the 4DCT DVFs, and finally application of the translated DVFs to the 4DFDK reconstructed volumes. Collectively, these steps required ∼2 min on our system without any effort to minimize computation time.

Results suggest that motion‐compensated reconstruction of rapid acquisition thoracic CBCT data may be feasible when two conditions are satisfied: (1) the phase bin is known for each projection and (2) the sparse angular sampling problem can be overcome, such as by using all projections in the reconstruction of each phase volume. When (1) and (2) are satisfied, we have shown MCFDK reconstruction quality to be comparable to that achievable with standard 4DFDK reconstruction of conventional acquisition CBCT data.

Several methods exist to determine the phase bin for clinically‐acquired CBCT projections. The Amsterdam shroud method^24^ exploits the sharp change in contrast between the diaphragm and lung to extract the phase bin directly from the projection data. Commercial systems use external imaging to monitor breathing and independently extract phase information during the scan. Examples include the Real‐Time Position Management system (Varian Medical Systems, Palo Alto, CA), the Active Breathing Coordinator (Elekta AB, Stockholm, Sweden), AlignRT (Vision RT Inc., London, UK), Catalyst HD (C‐RAD, Uppsala, Sweden), ExacTrac (Brainlab, Feldkirchen, Germany) and Identify (Varian Medical Systems, Palo Alto, CA).

The next phase of this work will apply our rapid acquisition MCFDK workflow on 4DCBCT data acquired using a clinical system. This will answer further questions about the clinical utility of rapid 4DCBCT in IGRT, such as whether rapid pre‐treatment scans capture motion that is truly representative of the motion present during treatment. Alternatively, do they make us more susceptible to short term variations in patient breathing? These areas require further investigation.

## 5 CONCLUSIONS

We used simulations of conventional and rapid acquisition kV CBCT to demonstrate that the projection data acquired during a rapid scan for 3D imaging in lung cancer radiotherapy may be suitable for motion‐compensated image reconstruction. Furthermore, the quality of rapid acquisition 4DCBCT images reconstructed with motion compensation may be comparable to that of current conventional 4DCBCT images.

## AUTHOR CONTRIBUTIONS

SJB, OD, HLB, and RTO contributed substantially to the study design and interpretation of data, drafted and revised this manuscript, have approved this version for publication and agree to be accountable for all aspects of this work. SJB and OD made further contributions on data acquisition and analysis.

## CONFLICT OF INTEREST

The authors have no relevant conflicts of interest to disclose.
